# Breast and bowel cancer screening uptake patterns over 15 years for UK south Asian ethnic minority populations, corrected for differences in socio-demographic characteristics

**DOI:** 10.1186/1471-2458-8-346

**Published:** 2008-10-02

**Authors:** Ala Szczepura, Charlotte Price, Anil Gumber

**Affiliations:** 1 Clinical Sciences Institute, Warwick Medical School, University of Warwick, Coventry, UK

## Abstract

**Background:**

A number of studies have reported low uptake of cancer screening programmes by South Asian populations in the UK. However, studies to date have not adjusted findings for differences in demographics and socio-economic status of these populations.

**Methods:**

**Subjects: **All residents in Coventry and Warwickshire, UK, eligible for screening. Uptakes compared for round 1 (2000–02) and round 2 (2003–05) of a national bowel cancer screening pilot, and for rounds 1, 2 and 5 of the established NHS breast cancer screening programme (commenced 1989).

**Data: **Bowel screening data were analysed for 123,367 invitees in round 1 and 116,773 in round 2 (total 240,140 cases). Breast screening data were analysed for 61,934, 62,829 and 86,749 invitees in rounds 1, 2 and 5 respectively (total 211,512 cases).

**Analysis: **Screening uptake was compared for two broad meta-categories (South Asian and non-Asian) and for five Asian subgroups (Hindu-Gujarati; Hindu-Other; Muslim; Sikh; South Asian Other). Univariate and multivariate analyses examined screening uptake and various demographic attributes of invitees, including age, gender, deprivation and ethnic group.

**Results:**

South Asians demonstrated significantly lower (*p *< 0.001) unadjusted bowel screening uptake; 32.8% vs. 61.3% for non-Asians (round 1). Rates were particularly low for the Muslim subgroup: 26.1% (round 1), 21.5% (round 2). For breast screening, a smaller difference was observed between South Asians and non-Asians; initially 60.8% vs. 75.4% (round 1) and later 66.8% vs. 77.7% (round 5). Thus, the disparity reduced gradually over time, alongside an overall trend of increased uptake. However, figures remained consistently low for Muslims (51% in rounds 1 and 5). After adjusting for age, deprivation (and gender), bowel screening uptake remained significantly lower for all South Asian subgroups. After similar adjustments, breast screening uptake remained lower for all subgroups except Hindu-Gujaratis.

For Muslims registered with an Asian (vs. non-Asian) GP, bowel screening uptake was significantly lower (*p *< 0.001). However, breast screening uptake for Muslims with an Asian (vs. non-Asian) GP showed no difference (*p *= 0.12) in the same period.

Colonoscopy and breast assessment uptakes were similar for both meta-categories, but Asian response time appeared slower for colonoscopy. The percentage of abnormal FOBT results was significantly higher for South Asian invitees. A slight increase in abnormal mammograms was observed for Muslims over time (2.7% to 4.2% in rounds 1 and 5 respectively).

**Conclusion:**

The lower cancer screening uptakes observed for the South Asian population cannot be attributed to socio-economic, age or gender population differences. Although breast screening disparities have reduced over time, significant differences remain. We conclude that both programmes need to implement and assess interventions to reduce such differences.

## Background

Breast cancer is the most common cancer in women in the United Kingdom (UK); bowel cancer is the second most common, and the third most common cancer in men after prostate and lung cancer [1]. Five year survival figures for bowel cancer are on average below 50% with poor survival largely attributable to late detection of the disease [2]. In contrast, five year survival for women diagnosed with breast cancer is 76% [3] and for cancers detected by screening 93% [4]. A population screening programme for breast cancer, first introduced nationally in 1988, is now well established in the UK.

Mammography uptake is approximately 71% at first invitation, with values ranging from 55% in London to 76% in other regions [5]. In 2000, the Department of Health set up pilot site areas to assess the feasibility of introducing a population screening programme for bowel cancer using faecal occult blood test (FOBT) kits completed at home and returned to a laboratory for processing [6]. An overall uptake level of 60% was reported in the pilot [7].

A number of international studies have highlighted inequalities in access to cancer screening for people from black and minority ethnic communities. In the UK, low uptake levels for breast screening have been observed, especially among South Asians [8-10]. In the United States (US), low breast screening rates have been reported for African-American women [11]. Although differences have narrowed over time [12-14], rates for Hispanic women appear to have remained lower than those for other groups [15]. For bowel cancer screening, the literature also provides evidence of lower uptake by ethnic minority communities. In the US, differences have been reported both for established ethnic minority groups such as African Americans [16-19] and for more recent immigrant Asian populations including Koreans, Japanese, Chinese and South-East Asians [20-22]. Low levels of bowel cancer screening have also been reported for immigrant populations in Europe [23]. In the UK, the impact of population diversity on uptake of cancer screening was not assessed in randomised controlled trials of FOBT screening [24] or flexible sigmoidoscopy screening [25]. Studies which compare breast and bowel cancer screening behaviour in the same population are limited; US research has reported that uptake is far lower for bowel screening than for breast screening among African American and Hispanic women [15]. Unfortunately, most studies fail to correct observed uptake differences for deprivation, so the possibility of socio-economic status acting as a confounding factor cannot be ruled out [26]. One study which has addressed this issue found that deprivation could not fully explain observed differences in uptake for bowel cancer screening, especially for older US ethnic minority populations [15].

The present study has analysed uptake patterns in a common UK population for two cancer screening programmes over time: breast screening (beginning with round 1 in 1989) and bowel cancer screening which started in 2000. Uptake patterns for South Asian minority groups have been compared to those for the majority population, adjusted for differences in demographics and socio-economic status. The research was funded by the National Health Service (NHS) Cancer Screening Programmes and the project was awarded Coventry Research Ethics Committee approval (ref: 05/Q2802/2) on 27th January 2005.

## Methods

### Setting

The study was undertaken in the English bowel cancer screening pilot site (Coventry and Warwickshire). This area has a population of over 800,000, including 8.7% ethnic minority residents who are mainly of South Asian origin. For breast screening all women aged 50–70 years registered with a general practitioner (GP) in the area are invited for mammography. The letter of invitation, patient information leaflet explaining the importance of breast cancer screening, and any reminders are all printed in English, although GP practices do display patient information leaflets in various languages. For bowel cancer screening all men and women aged 50–69 years registered with a GP are invited. For this programme, the invitation letter contains a sentence in eleven languages (including the main South Asian ones) explaining how a translated patient information leaflet can be obtained. Instruction leaflets explaining how to use the FOBT kit are in English.

### Data Preparation

Data were downloaded from the records of the two cancer screening programmes (Warwickshire, Solihull & Coventry Breast Screening Service; and the English Bowel Cancer Screening Pilot, Coventry & Warwickshire). Selected items were collated for all individuals invited to either cancer screening programme. Breast cancer screening data were obtained for round 1 (1989 – 1992), round 2 (1992 – 1995) and round 5 (2001 – 2004). Bowel screening data for round 1 (2000 – 2002) and round 2 (2003 – 2005). Data items extracted included demographic descriptors (age, sex, postcode of residence), screening invitation date, GP details, screening outcome, and final diagnosis. For bowel cancer, screening outcomes included: individual's response to home testing invitation; number of FOBT kits completed; screening test result; response to colonoscopy invitation if FOBT positive; and diagnostic outcome. For breast cancer, screening outcomes included: response to mammography invitation; mammography result; response to assessment invitation if an irregularity is found in the mammogram; and diagnostic outcome. To ensure reliability, individuals were removed from the data files if: excluded from screening by the Health Authority e.g. address not found; individual undergoing treatment or recently deceased; outside the specified age range; or received a second invitation within the screening round period (in which case only the earliest record was retained).

#### Deprivation

The South Asian population in the study area is concentrated in distinct locations. The postcode of residence was used to link UK Census data on deprivation from the Census Dissemination Unit (MIMAS, University of Manchester, England) to individuals. Linkage was undertaken at electoral ward level for the 1991 Census and at Central Area Statistic ward level for the 2001 Census; these areas are designed to be homogeneous in socio-economic characteristics. The Carstairs Index of Deprivation, with cut-off values for England, was used as the indicator of deprivation [7]. 1991 deprivation scores were used for rounds 1 and 2 of the breast screening programme; 2001 scores were used for later breast screening (round 5) and both bowel screening rounds.

#### Ethnicity

Ethnicity is poorly recorded in the UK, especially in primary care [27-29]. This frequently precludes even the most basic analysis of inequalities in access to services [30-33]. The present study used name recognition software which offers a useful alternative for identification of populations with distinctive names, such as South Asians [34-36]. The software was validated on local name datasets containing (gold standard) self-assigned ethnicity; this demonstrated sensitivity/specificity values of 95% and 97% respectively [37,38]. Further refinement using manual checking by experts of the 180,000 names assigned by the software produced an estimated final sensitivity of 97%. The software was used to assign invitees to the following religio-linguistic groups: Hindu-Gujarati; Hindu-Other; Muslim; Sikh; South Asian Other; non-Asian. The meta-category 'South Asian' refers to the first five groups combined. The ethnic origin of GPs was determined using the same software.

### Analysis

For breast screening, uptake levels were compared at two stages in the screening process: (i) attendance for mammography in response to a routine screening invitation; (ii) attendance for further assessment if an irregularity was found in the mammogram. For bowel cancer screening, uptake levels were compared at three stages: (i) return of an initial FOBT kit, even if this proved to be inadequately completed; (ii) successful completion of a home FOBT kit; (iii) for those with a positive FOBT result, attendance for a colonoscopy appointment. Cases were excluded if medical unfitness or other legitimate explanations were recorded as reasons for failure to perform the colonoscopy.

Logistic regression was used to explore associations between levels of screening uptake and various demographic attributes of invitees. Both unadjusted and adjusted odds ratios (point estimates and 95% confidence intervals) were calculated. The adjusted analyses were used to control uptake in different ethnic groups for factors such as gender (bowel screening only), age and deprivation. Age was categorised into four bands (50–54; 55–59; 60–64; 65–69) and deprivation into five bands.

The study also explored the possible influence of GP characteristics, with a particular focus on ethnicity. For breast screening this analysis was undertaken only on round 5 data, since historical information on GPs was unavailable for rounds 1 and 2.

## Results

**Figure 1:**
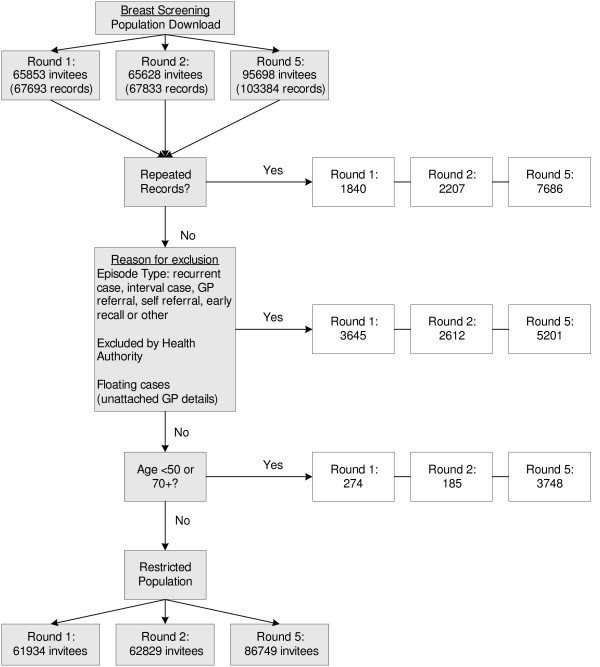
Flowchart detailing process for obtaining breast screening populations for analysis.

**Figure 2:**
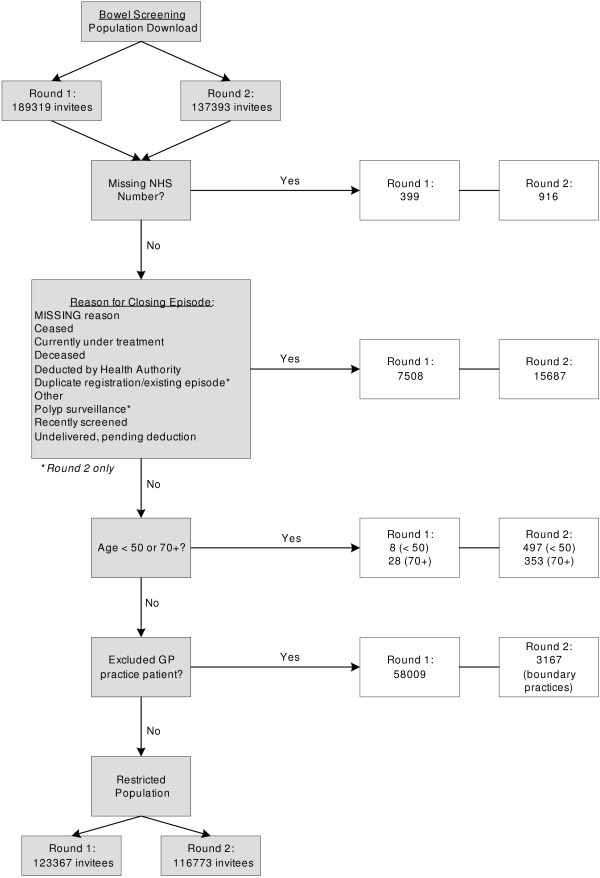
Flowchart detailing process for obtaining bowel screening populations for analysis.

Figures 1 and 2 present flowcharts showing the final populations included in analysis of breast and bowel cancer screening datasets respectively. In 0.5% of bowel and 0.7% of breast invitees a deprivation index could not be computed due to missing postcode data. The religio-linguistic indicator could be attached to all except 2 cases.

### Relationship between screening uptake and invitee characteristics

Breast screening uptake for the South Asian population was significantly lower than for non-Asians in all 3 rounds. In round 1, the observed uptake was 60.8% for South Asians compared to 75.4% for non-Asians; giving a difference of 14.6% (95% confidence interval (CI): 12.6 to 16.5). In round 2, the difference was 12.6% (95% CI: 10.8 to 14.4) and in round 5 it was 10.9% (95% CI: 9.4 to 12.3). Thus, there is evidence that the disparity in breast screening uptake is reducing gradually over time. For bowel screening, the observed differences are larger than for breast screening. In round 1, the South Asians' completion rate of 32.8% was approximately half of that for non-Asians (61.3%), giving a difference of 28.5% (95% CI: 27.3 to 29.6). Figures for round 2 were 29.6% for South Asians compared to 55.8% for non-Asians, giving a difference of 26.2% (95% CI: 25.2 to 27.3).

When uptake levels are examined at the subgroup level, further differences emerge. For breast screening, Hindu-Gujarati women exhibited the highest initial uptake (round 1). This has increased over time so that by round 5 their uptake was not significantly different from that of non-Asian women (*p *= 0.19). Breast screening uptake has failed to improve for only one subgroup (Muslim women). For bowel cancer screening, in contrast, uptake levels have decreased slightly between rounds for all groups. However, once again, Muslim invitees have the lowest levels of FOBT completion and the Hindu-Gujarati population the highest. Hindu-Gujaratis are the only subgroup to show a (slight) narrowing of the gap with non-Asian invitees. When compared to the uptake for all other South Asian subgroups combined, Muslims exhibit a significantly lower bowel screening uptake in both rounds (*p *< 0.001).

**Figure 3:**
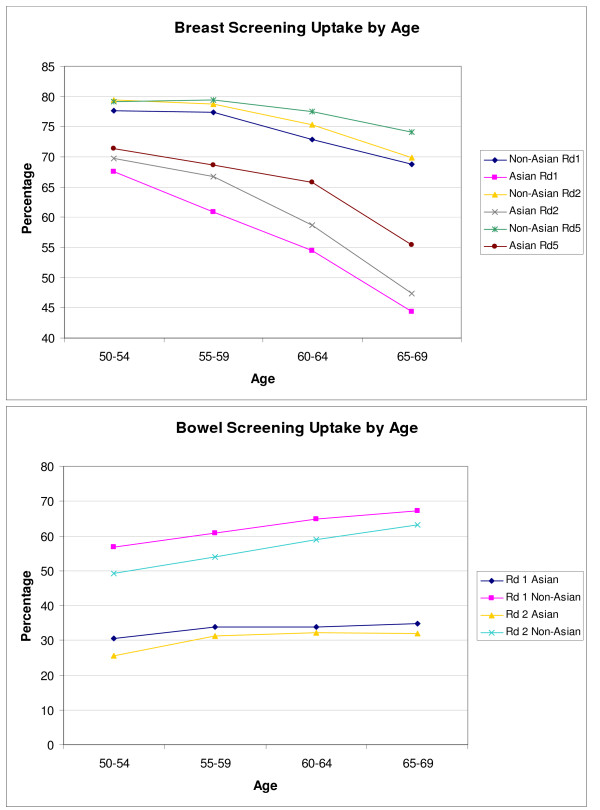
Breast and bowel screening uptake by age group.

Figure 3 shows that breast screening uptake demonstrates a decrease with age in all three rounds for both South Asians and non-Asians (*p *< 0.001). For bowel cancer screening, there is an increased uptake with age for the non-Asian population in both rounds (*p *< 0.001). For South Asians, however, the effect of age is less marked and above the 55–59 year age group there is no significant increase (*p *= 0.53 and *p *= 0.71 for rounds 1 and 2 respectively).

In the bowel screening programme, the gender balance of invitees from the South Asian and non-Asian communities was identical, at 50% (± 0.2%) males. FOBT uptake was generally lower for males than for females in both groups, although this difference in the South Asian population is much smaller. In round 1, uptake by non-Asian males (56.6%) was significantly lower than for females (66.0%); a difference of 9.4% (95% CI: 8.8 to 9.9). Similarly in round 2, the male figure was 51.5% and the difference observed was 8.6% (95% CI: 7.0 to 9.2). In contrast, for the South Asian population, gender differences are less significant; in round 1, a difference of 1.1% (95% CI: -1.2 to 3.3), and in round 2 a difference of 1.5% (95% CI: -0.5 to 3.6).

**Figure 4:**
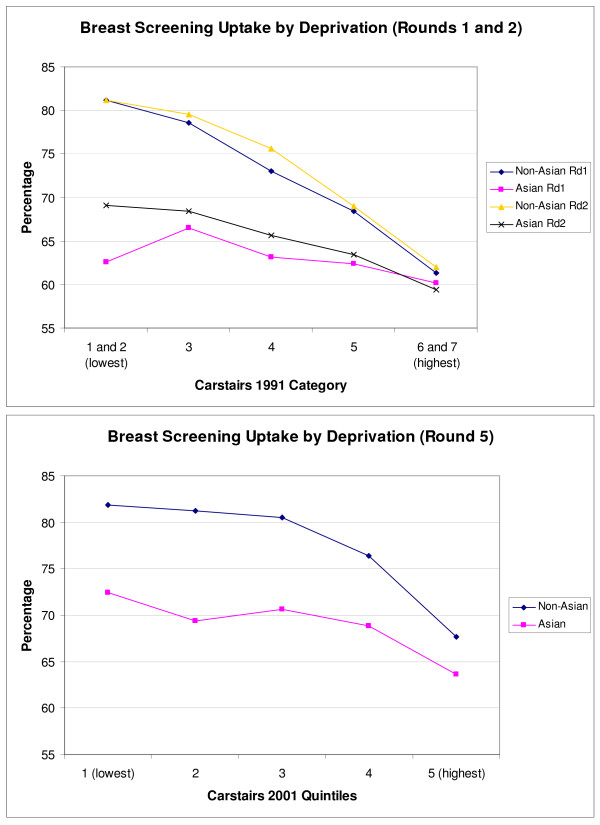
Breast screening uptake by deprivation group.

**Figure 5:**
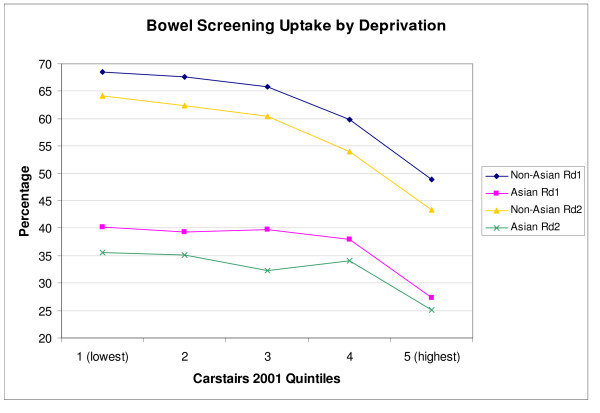
Bowel screening uptake by deprivation group.

The influence of socio-economic status on uptake of breast screening is shown in Figure 4. For non-Asian invitees, uptake clearly decreases with increased deprivation in all 3 rounds (*p *< 0.001). This pattern appears less pronounced for South Asian invitees, with a decrease only evident in later rounds (*p *≤ 0.001 rounds 2 and 5). In the most deprived group, there is no evidence of a difference in uptake between South Asian and non-Asian invitees in round 1 (*p *= 0.60) or round 2 (*p *= 0.24), although a significant difference is apparent in round 5 (*p *< 0.001). For bowel cancer screening, Figure 5 shows that uptake once again decreases with increased deprivation for non-Asian invitees in both rounds (*p *< 0.001). For the South Asian invitees, on the other hand, uptake remains relatively constant apart from a decrease for the most deprived group in both rounds. Overall, the strength of any link between deprivation and screening uptake is less evident in the South Asian population for both screening programmes.

### Multivariate analysis

Tables 1, 2, 3 show the results from the main effects logistic regression model, including adjusted odds ratios with corresponding 95% confidence intervals. For breast screening, three invitee characteristics were included (age, deprivation and ethnicity); for bowel screening gender was also included in the regression model. Multivariate analyses excluded the 'South Asian Other' group due to low numbers.

**Table 1:** Multivariate analysis: breast and bowel screening uptakes by age group and gender

**Cancer screening Programme/Round**	**Age**	**Number**	**Uptake (%)**	**Adjusted OR (95% CI)**
	50–54	17453	77.13	1 (-)
	55–59	20307	76.63	0.97^+^ (0.92, 1.02)
Breast screening	60–64	20081	72.16	0.78** (0.74, 0.82)
Round 1: 1989–92	65–69	4051	68.28	0.65** (0.60, 0.71)
	50–54	19176	78.99	1 (-)
	55–59	20894	78.15	0.96^+^ (0.91, 1.01)
Breast screening	60–64	19144	74.68	0.79** (0.75, 0.83)
Round 2: 1992–95	65–69	3577	69.33	0.61** (0.56, 0.67)
	50–54	26909	78.71	1 (-)
	55–59	26175	78.93	1.00^+^ (0.96, 1.04)
Breast screening	60–64	19733	76.93	0.91** (0.87, 0.95)
Round 5: 2001–04	65–69	13394	73.32	0.75** (0.71, 0.79)
	50 – 54	36832	54.74	1 (-)
	55 – 59	34931	29.30	1.18** (1.15, 1.22)
Bowel screening	60 – 64	27518	62.58	1.41** (1.36, 1.45)
Round 1: 2000–02	65 – 69	23968	64.86	1.56** (1.51, 1.61)
	50 – 54	32426	47.44	1 (-)
	55 – 59	34309	52.88	1.21** (1.17, 1.24)
Bowel screening	60 – 64	27336	57.69	1.49** (1.44, 1.54)
Round 2: 2003–05	65 – 69	22588	61.44	1.77** (1.71, 1.84)
**Cancer screening Programme/Round**	**Gender**	**Number**	**Uptake (%)**	**Adjusted OR (95% CI)**
Bowel screening	Male	61650	55.30	1 (-)
Round 1: 2000–02	Female	61599	64.21	1.46** (1.43, 1.50)
Bowel screening	Male	58395	50.08	1 (-)
	
Round 2: 2003–05	Female	58264	58.21	1.39** (1.36, 1.43)

^+^Non-significant *p *> 0.05

** Significant at *p *< 0.001

**Table 2:** Multivariate analysis: breast and bowel screening uptakes by deprivation

**Cancer screening Programme/Round**	**Deprivation**	**Number**	**Uptake (%)**	**Adjusted OR (95% CI)**
	1 & 2 (Least)	15535	80.86	1 (-)
	3	17387	78.35	0.87** (0.82, 0.91)
Breast screening	4	15894	72.54	0.64** (0.61, 0.68)
Round 1: 1989–92	5	5224	68.09	0.53** (0.49, 0.56)
	6 & 7 (Most)	3193	61.13	0.41** (0.38, 0.45)
	1 & 2 (Least)	17374	80.99	1 (-)
	3	18390	79.28	0.91** (0.86, 0.95)
Breast screening	4	15847	75.12	0.73** (0.69, 0.77)
Round 2: 1992–95	5	5051	68.62	0.53** (0.50, 0.57)
	6 & 7 (Most)	3020	61.39	0.42** (0.38, 0.45)
	1 (Least)	16903	82.18	1 (-)
	2	17002	81.27	0.95^+^ (0.90, 1.00)
Breast screening	3	17228	80.50	0.91** (0.86, 0.96)
Round 5: 2001–04	4	17117	76.37	0.72** (0.68, 0.76)
	5 (Most)	17351	67.60	0.48** (0.45, 0.50)
	1 (Least)	16107	67.78	1 (-)
	2	23420	66.95	0.96* (0.92, 1.00)
Bowel screening	3	25225	65.03	0.88** (0.84, 0.91)
Round 1: 2000–02	4	28301	58.68	0.68** (0.66, 0.71)
	5 (Most)	29855	46.34	0.44** (0.42, 0.45)
	1 (Least)	14444	63.18	1 (-)
	2	21831	61.45	0.92** (0.88, 0.96)
Bowel screening	3	24090	59.55	0.84** (0.81, 0.88)
Round 2: 2003–05	4	26852	52.80	0.66** (0.63, 0.68)
	
	5 (Most)	28823	40.98	0.43** (0.41, 0.45)

+ Non-significant *p *> 0.05

* Significant at p < 0.05

** Significant at *p *< 0.001

**Table 3:** Multivariate analysis: breast and bowel screening uptakes by ethnic subgroup

**Cancer screening Programme/Round**	**Ethnic Group**	**Number**	**Uptake (%)**	**Adjusted OR◆ (95% CI)**
	Hindu-Gujarati	477	67.71	0.93^+ ^(0.75, 1.15)
	Hindu Other	241	59.75	0.60** (0.45, 0.79)
Breast screening	Muslim	567	51.32	0.49** (0.41, 0.58)
Round 1: 1989–92	Sikh	1245	63.37	0.67** (0.59, 0.76)
	Non-Asian	59362	75.35	1 (-)
	Hindu-Gujarati	522	69.73	0.86^+ ^(0.71, 1.05)
	Hindu Other	247	67.21	0.67** (0.51, 0.89)
Breast screening	Muslim	582	53.26	0.46** (0.39, 0.54)
Round 2: 1992–95	Sikh	1313	67.56	0.69** (0.61, 0.78)
	Non-Asian	60127	77.38	1 (-)
	Hindu-Gujarati	758	75.86	1.13^+ ^(0.95, 1.34)
	Hindu Other	428	68.93	0.68** (0.56, 0.84)
Breast screening	Muslim	912	51.75	0.40** (0.35, 0.46)
Round 5: 2001–04	Sikh	2067	70.59	0.79** (0.72, 0.88)
	Non-Asian	82046	78.05	1 (-)
	Hindu-Gujarati	1389	40.03	0.50** (0.45, 0.56)
	Hindu-Other	681	34.51	0.38** (0.32, 0.44)
Bowel screening	Muslim	1595	26.14	0.30** (0.27, 0.34)
Round 1: 2000–02	Sikh	3012	32.47	0.36** (0.33, 0.39)
	Non-Asian	116572	61.30	1 (-)
	Hindu-Gujarati	1478	37.01	0.55** (0.50, 0.62)
	Hindu-Other	757	33.16	0.44** (0.38, 0.52)
Bowel screening	Muslim	1807	21.47	0.29** (0.26, 0.33)
Round 2: 2003–05	Sikh	3378	29.66	0.39** (0.36, 0.42)
	
	Non-Asian	109239	55.83	1 (-)

◆ Controlled for age, deprivation, gender (breast screening)

^+^Non-significant *p *> 0.05

*Significant at p < 0.05

**Significant at *p *< 0.001

For breast screening, the adjusted odds ratio for the total population decreases with increasing age in all three rounds (Table 1), indicating that older women are less likely to undertake screening, even if their other characteristics such as deprivation and ethnicity are taken into account. Similarly, deprivation has a very strong influence on breast screening behaviour in all 3 rounds (Table 2), with women in the highest deprivation category two or three times less likely to undertake screening than women in the least deprived category, particularly in rounds 1 and 2. For bowel cancer screening, Table 1 shows that, in contrast to breast screening, uptake levels increase with age. The adjusted odds of undertaking screening are over 1.5 times higher in the oldest age group compared to the youngest reference age group. Adjusted odds are also higher for the female population in both rounds (Table 1), although differences appear to be slightly lower in round 2 than in round 1. As is observed for breast screening, uptake decreases with increased deprivation in both rounds (Table 2), with the odds of undertaking screening, after adjusting for age, gender and ethnicity, less than half for invitees in the most deprived group.

The major uptake differences are linked to ethnicity (Table 3). For bowel screening, even after adjusting for age, gender and socio-economic status, there are clear differences between those in South Asian subgroups compared to the non-Asian reference group. In particular, the adjusted odds ratios show that the likelihood that a Muslim invitee will successfully complete the FOBT home screening test is approximately one third that of a non-Asian invitee. Even for the ethnic subgroup with the highest uptake (Hindu-Gujarati), the adjusted odds are 0.5. It appears that lower bowel cancer screening uptake levels in the South Asian population cannot be explained by differences in other characteristics such as deprivation. The remaining two groups (Sikh and Hindu-Other) demonstrate lower uptake of bowel screening compared to the non-Asian reference group, although differences are not as marked as those seen in the Muslim population. Similarly, for breast screening the adjusted odds ratios indicate significant differences, with lower screening uptake among South Asian invitees. Muslim women show a decrease in the adjusted odds ratio over rounds 1 to 5, while adjusted odds ratios for the Hindu-Gujarati population are not significantly different to those for the non-Asian population. Once again, Sikh and Hindu-Other groups demonstrate lower uptakes compared to the non-Asian and Hindu-Gujarati groups, although not as low as those observed for Muslim women.

### Screening uptake and GP characteristics

Nearly two thirds of South Asian women invited to undertake breast screening are registered with a South Asian GP; a similar pattern is observable at the ethnic subgroup level. Differences in breast screening uptake for those registered with a South Asian vs. non-Asian GP are largest for Muslim women (49.4% vs. 54.9% respectively), although this difference is not significant (*p *= 0.12). For bowel cancer screening, however, much larger differences in uptake are observed, especially for the Muslim population. Analysis confirms that there are significant (*p *< 0.001) differences for Muslims in round 1 (22.9% for women registered with a South Asian GP vs. 38.0% for those registered with a non-Asian GP), giving a difference of 15.1% (95% CI: 9.6 to 20.8); and for round 2 (*p *< 0.001) with figures of 19.2% and 27.4% respectively, giving a difference of 8.2% (95% CI: 3.8 to 12.8).

### Screening and diagnostic outcomes

Although irregularities were found to be more likely in non-Asian mammograms than for South Asian women in all 3 rounds, these differences were not significant (*p *> 0.1). In contrast, for bowel screening in both rounds the percentage of abnormal FOBT results was significantly higher for South Asian invitees; 3.1% (95% CI: 2.4 to 3.9) in round 1, and 4.7% (95% CI: 3.9 to 5.7) in round 2 vs. 1.2% and 1.4% respectively for non-Asians. Following an irregularity in the mammogram, all women attended for further assessment regardless of their ethnicity. Similarly, colonoscopy uptake rates were identical at 6 months, regardless of ethnicity. However, there is evidence of some delay for the South Asian patients, which has increased over rounds. In round 1, 85% of South Asian patients as opposed to 90% of non-Asian patients undertook colonoscopy within 3 months of obtaining the abnormal FOBT result. In round 2, comparable figures were 79% for South Asians and 89% for non-Asians. (*p *= 0.05).

Following breast assessment, a higher percentage of abnormal results are recorded among South Asians, although this difference is not statistically significant (p > 0.1 in all rounds). Following colonoscopy, the likelihood of detecting bowel cancer is not significantly different between South Asians and non-Asians. It should be noted, however, that numbers in both cases are very low.

## Discussion

In the UK, various policy documents [39,40] and the legal requirements set out in the Race Relations (Amendment) Act 2000 [41] have all called for a change from the concept of 'average citizen' to one that recognises diversity. A recent review of 'ethnic issues in screening' produced by the NHS National Screening Committee [42] has recommended that all nationally managed screening programmes ensure effective demographic data collection (including data on ethnicity) and assess the likely impact of any new policy on the promotion of race equality.

The findings presented in this paper are important because they represent the first body of systematic research evidence based on individual patients in different South Asian populations. This provides robust evidence of disparities in screening uptake for existing and new cancer screening programmes even after adjusting for differences in demographics and socio-economic status. With nearly one in ten UK citizens from a minority ethnic background, improved understanding of how the offer of cancer screening is received by different populations is an increasingly important issue [43,44]. Our findings support evidence from earlier analyses based on practice-level data, indicating that cancer screening uptake might be significantly lower in certain ethnic minority populations such as South Asians [8].

Our analysis over screening rounds shows that breast screening uptake has improved for the South Asian population at a faster rate than for the majority population, with the net result that differences between the two populations have reduced significantly from 14.6% in 1989–92 to 10.9% in 2003–05, although a significant disparity remains. Our findings are similar to those from the US where differences in mammography uptake are reported to have narrowed over time [12-14]; this decrease has occurred against the background of a general increase in breast screening uptake rates for eligible women [45], similar to the pattern we identify, and of lower breast screening uptakes by women of African-American, Hispanic and Native-American origin [11,46-51]. The difference we report is however smaller than the 78% vs. 53% disparity reported by another 2001 UK study [52], although this study was undertaken in an area with 61% Muslims in the South Asian population [53]. At the subgroup level, our findings demonstrate that the Hindu-Gujarati population achieved parity with the majority population over the period up to 2005, while Muslim women are the only group for whom breast screening uptake has not improved over time. A slight increase in abnormal mammograms was observed for Muslims over time (2.7% to 4.2% in rounds 1 and 5 respectively). It is interesting to note that uptake rates for Hispanic women also appear to have remained lower than for other groups such as African-American or Caucasian women in the US [15]. Although our findings indicate that breast screening uptake has improved for all age groups over time in both populations, South Asian women, especially older women, continue to exhibit a significantly lower uptake. In the US, mammography uptake is similarly reported to fall with age; the regularity with which women undertake screening also varies, with older women, African-Americans and those from more deprived populations showing more infrequent use [11,12]. In our study a slight increase in abnormal mammograms was observed for Muslims over time which might be linked to less frequent screening use.

Our findings indicate a general pattern of lower breast screening uptake in more deprived groups for both South Asian and non-South Asian populations, although the effect is far less pronounced for South Asians. Most importantly, our multivariate analysis shows that lower breast screening uptake rates in the South Asian population cannot be explained by factors such as deprivation. At a GP practice level, other researchers have identified social deprivation and ethnic-mix in the local population as correlated with breast screening rates in the UK [54]. However, the possible confounding effect of deprivation has generally not been separated from that of ethnicity. In the US, various socio-economic characteristics e.g. income, education, insurance status appear to characterise populations with lower rates of breast cancer screening, with some research evidence emerging to indicate that ethnic disparities in uptake remain even after allowing for socio-economic differences [11,55-57].

For bowel cancer screening, our results show that the global uptake level achieved (60%) masks significantly lower uptake rates for South Asians; differences are even greater than those observed for breast screening. Both men and women from the South Asian community are far less likely to return an initial home test kit or to subsequently successfully complete the testing process, than are non-Asians. Evidence from other countries demonstrates a similarly low uptake of bowel cancer screening (FOBT, colonoscopy and flexible sigmoidoscopy) by ethnic minority populations [15-19,23,58-60]. Studies of American Asian immigrant populations e.g. Koreans and Vietnamese report similar findings [20-22,61]. Once again, very few of these studies have controlled uptake for differences in socio-economic status, but those that have report inconsistent findings [15,58,59]. The present study provides clear evidence that 'ethnicity', although it may correlate with socio-economic status, exerts a separate effect on response to bowel cancer screening. There is also evidence of some delay in the South Asian population before undertaking colonoscopy.

The findings reported in this paper add to the developing evidence base on disparities in access to a range of services by ethnic minorities [62,63]. It might be anticipated that bowel cancer screening would present greater barriers for ethnic minorities than breast screening. Home FOBT testing requires that individuals not only understand the benefits of screening, but also that they are able to follow specific written instructions in order to collect and preserve samples over a number of days. A separate analysis of round 1 bowel screening data indicates that South Asians who attempt an initial home testing are more likely to be sent 4 or more kits before successful completion (e.g. 28% of Muslims compared with only 3% of non-Asians) [54]. South Asian women are found to exhibit particularly low FOBT completion rates compared to their non-Asian peers, especially among older women. This may be linked to the provision of written materials, including kit instructions, since Muslim and older South Asian women are known to have particularly low literacy levels [64,65].

The ethnicity of the GP also appeared to be associated with lower uptake of bowel cancer screening by South Asians in our study. The same effect was not observed for breast screening. It is difficult to see why this should be the case since GPs are not directly involved in the bowel screening process. However, studies in other health care systems have identified physician recommendation as a significant predictor of FOBT uptake by ethnic minorities [8,61,66,67].

The implications of our findings for roll-out of the UK bowel cancer screening programme will be especially significant for inner city areas where South Asian populations can reach 40% [53]. In order to ensure equity as well as efficiency in the new bowel cancer screening programme, this population will require special attention. Interventions targeted at particular groups may be needed in order to achieve more equitable uptake rates e.g. Muslims or older South Asian women. At present there is limited research evidence to indicate what types of intervention would prove most effective [68-71]. Equally importantly, the continued disparity in breast screening uptakes after two decades needs to be addressed. A recent review of the literature on interventions to improve breast screening uptake in diverse populations suggests that combined approaches using access-enhancing and individual-directed strategies are most effective [72].

Finally, it might be argued that the incidence of cancer among South Asian populations in the UK is low, and that the observed differences in screening uptake are therefore relatively unimportant. However, the low cancer incidences reported historically are increasingly seen as an artefact of the stage of migration with reports of increased incidence beginning to emerge [73-75]. One limitation of the present study is that we are unable to draw any conclusions about the African Caribbean population because of incomplete ethnic monitoring data and the fact that name recognition software cannot identify these individuals. However, there was a relatively small population in the study area (< 1%). The need to urgently improve routine ethnic data collection to provide improved statistics on cancer incidence and survival for all ethnic minority groups in the UK has recently been highlighted [76].

## Conclusion

We conclude that the low breast and bowel cancer screening uptake rates observed in the South Asian population cannot be attributed to socio-economic or age/gender population differences. Although disparities in breast screening have reduced over time, they are still significant. We would suggest that both programmes need to identify and assess culturally appropriate interventions to reduce these observed differences, including provision of tailored health promotion materials for certain South Asian subgroups. It would appear that Muslim invitees registered with a South Asian GP are a prime target for improved bowel screening uptake. More detailed examination of behaviour across the two screening programmes may also help to identify women who have responded positively to breast screening and could therefore be encouraged to complete bowel screening successfully.

## Competing interests

The authors declare that they have no competing interests.

## Pre-publication history

The pre-publication history for this paper can be accessed here:


